# Spin-flip-driven anomalous Hall effect and anisotropic magnetoresistance in a layered Ising antiferromagnet

**DOI:** 10.1038/s41598-023-30076-2

**Published:** 2023-02-28

**Authors:** Dong Gun Oh, Jong Hyuk Kim, Mi Kyung Kim, Ki Won Jeong, Hyun Jun Shin, Jae Min Hong, Jin Seok Kim, Kyungsun Moon, Nara Lee, Young Jai Choi

**Affiliations:** Department of Physics, Yonsei University, Seoul, 03722 Korea

**Keywords:** Electronic properties and materials, Magnetic properties and materials, Spintronics

## Abstract

The influence of magnetocrystalline anisotropy in antiferromagnets is evident in a spin flip or flop transition. Contrary to spin flops, a spin-flip transition has been scarcely presented due to its specific condition of relatively strong magnetocrystalline anisotropy and the role of spin-flips on anisotropic phenomena has not been investigated in detail. In this study, we present antiferromagnet-based functional properties on an itinerant Ising antiferromagnet Ca_0.9_Sr_0.1_Co_2_As_2_. In the presence of a rotating magnetic field, anomalous Hall conductivity and anisotropic magnetoresistance are demonstrated, the effects of which are maximized above the spin-flip transition. Moreover, a joint experimental and theoretical study is conducted to provide an efficient tool to identify various spin states, which can be useful in spin-processing functionalities.

## Introduction

Following recent observations in antiferromagnetic (AFM) spintronics revealing that an AFM order dominates dynamic transport through the system^1–3^, AFM materials have been considered as promising candidates for the future generation of spintronic technology. The fundamental feature for detecting a controlled AFM memory state is the magnetocrystalline anisotropy. Accordingly, electrical means such as anisotropic magnetoresistance (AMR) and anomalous Hall effect (AHE) have been adopted to detect the orientation of the Néel vector^4–13^. The influence of magnetocrystalline anisotropy on the magnetism is well exemplified in a spin-flop or flip transition. A spin-flop transition with a relatively weak magnetocrystalline anisotropy can be found in a wide range of AFM compounds, such as NiO, Li_2_MnO_3_, NiWO_4_, CsCo_2_Se_2_, MnBi_2_Te_4_, Cu_2_(OH)_3_Br, EuMnBi_2_, and Gd_5_Ge_4_^14–21^. A controlled anisotropic phenomenon through the transition supplies an essential understanding of elemental magnetism and broad spintronic applicability.

In contrast to spin-flops, a spin-flip transition has been seldom reported owing to its particular condition requiring relatively strong magnetocrystalline anisotropy. The magnetic properties of Ca_1-*x*_Sr_*x*_Co_2_As_2_ compounds are strongly dependent on chemical doping, which modifies the distance between magnetic layers, thereby inducing variations in the interlayer magnetic couplings and magnetocrystalline anisotropy. In a bare CaCo_2_As_2_ compound, a spin-flop transition occurs at *H*_flop_ = 3.7 T for *T* = 4 K^29^. In the present study, we selected the Ca_0.9_Sr_0.1_Co_2_As_2_ (CSCA) compound because its magnetic transition is optimized with a much sharper and more intact step-like shape compared to that of CaCo_2_As_2_^22,23^. In addition, the magnetic phase transition is lowered with increasing *T*_N_. Nonetheless, in the 20% Sr-doped compound (*x* = 0.2), the modified interlayer coupling gives rise to a complete phase change, resulting in a ferromagnetic (FM) state^24^. Another AFM phase with an easy *ab* plane has also been observed after further Sr-doping (*x* = 0.3)^24^. The sign variation in exchange coupling, which is sensitive to the *c*-axis parameter, is plausibly attributed to indirect Ruderman–Kittel–Kasuya–Yosida-like exchange interactions^28^.

In our previous work, we investigated anisotropic magnetic properties by combining angle dependence of magnetic torque measurement and easy-axis spin model calculation^25^. In this study, we explored anisotropic magneto-transport properties in AFM CSCA, wherein magnetic multilayers are intrinsically formed. The CSCA crystal revealed a field-tunable AHE in which the on–off behavior can be observed across the spin-flip transition. AMR was also found to be maximized in the vicinity of the flip transition. We expanded the spin model calculation to the estimation of the AMR by considering electron hopping between adjacent Co_2_As_2_ layers, composed of both spin-dependent and spin-independent parts. As a result, the intricate magneto-transport characteristics can be elucidated by the close correlation between the spin states and magnetoresistance/anomalous Hall conductivity data.

## Results and discussion

**Figure 1:**
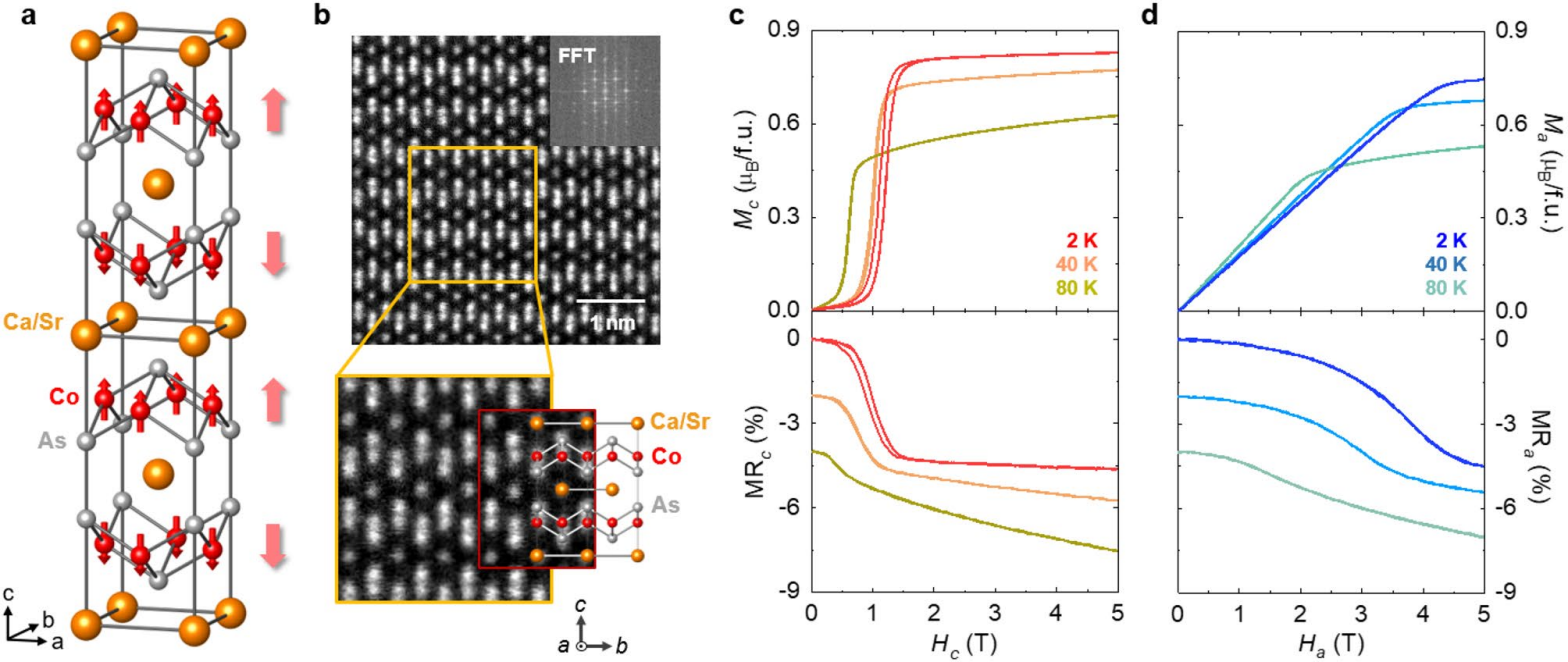
Structure and magnetic field-dependent magnetization and magnetoresistance. (**a**) Crystal structure of body-centered tetragonal Ca_0.9_Sr_0.1_Co_2_As_2_. The orange, red, and grey spheres represent Ca/Sr, Co, and As atoms, respectively. The small red arrows on the Co atoms represent the individual spin directions, whereas the large faded red arrows (on the right) indicate the net magnetic moments of the Co_2_As_2_ layers. (**b**) Scanning transmission electron microscopy (STEM) dark-field images recorded in the *bc* plane. The STEM image with lower magnification illustrates the regular alignment of all layers. The inset depicts the diffraction pattern obtained via fast Fourier transform. (**c**) Isothermal magnetization and magnetoresistance along the *c*-axis at *T* = 2, 40, and 80 K. Magnetoresistance data were shifted vertically to facilitate distinction. **(d**) Isothermal magnetization and magnetoresistance along the *a*-axis at *T* = 2, 40, and 80 K.

The CSCA crystal belongs to the ThCr_2_Si_2_-type structure family, which has been extensively investigated in the interest of versatile magnetic and electronic states^22,24^. Such compounds have received particular attention owing to their varying types of superconductivities, including Fe-based high-temperature (*T*) superconductivity in doped BaFe_2_As_2_^26,27^ and heavy fermion superconductivity in CeCu_2_Si_2_^28,29^; such compounds including BaNi_2_As_2_^30,31^, LaIr_2_Ge_2_^32^, and LaRu_2_P_2_^33^ consist of various transition metals. CSCA single crystals form a tetragonal structure (I4/mmm space group), where two Co_2_As_2_ layers lie opposite to each other around the center of a unit cell, separated by a non-magnetic (NM) Ca/Sr single layer, as depicted in Fig. 1a. This unit is stacked repeatedly along the *c*-axis. In our analysis, we used the single crystal X-ray diffraction technique to inspect the crystal quality, which is a crucial aspect in examining notable anisotropy. The analyses revealed high-quality single-phase crystals (see Supplementary S1). Co spins, which act as FM within a layer, are known to couple antiferromagnetically with the spins in the neighboring layer, resulting in a complete cancellation of the magnetic moments, i.e., the A-type AFM order (Fig. 1a)^22,24^. Herein, the structural units were elucidated using scanning transmission electron microscopy (STEM) (Fig. 1b). Further, the following lattice constants were determined via the application of the fast Fourier transform to the STEM data: *a* = 0.408 nm and *c* = 1.086 nm. A regular arrangement of the layers is clearly revealed in the low magnification STEM image. AFM order emerges at the Néel temperature, *T*_N_ ≈ 97 K, which represents an anomaly in the magnetic susceptibility and *T* derivative of resistivity (see Supplementary Fig. S2 for details).

Antiferromagnetism is generally explained via a collinear two-sublattice model^34,35^. In the absence of a magnetic field (*H*), two-sublattice magnetization (*M*) vectors with equal magnitude are aligned in opposite directions. A sufficiently strong *H* along the AFM spin axis may induce flops or flips of the *M* vectors, resulting in a magnetic transition. The primary feature of this transition is spin reorientation through phase conversion, which renders distinct anomalies in physical properties^36–39^. A large step-like increase in *M*_*c*_ (*M* along the *c*-axis) at *H*_flip_ = 1.2 T and *T* = 2 K results in a spin-flip transition, as presented in Fig. 1c. A noticeable magnetic hysteresis can be identified, reflecting a first-order nature of the transition. As *T* increases, *H*_flip_, determined by the *H*-derivative of *M*_*c*_, progressively decreases from 1.2 T at 2 K to 0.6 T at 80 K. The error bars obtained from the spin-flip transitions at various *T*s are plotted in Supplementary Fig. 4. In contrast, the gradual canting of Co spins in *M*_*a*_ (*M* along the *a*-axis) at 2 K leads to a linear increase up to 4.0 T, above which the slope of *M*_*a*_ considerably declines (Fig. 1d). The *H* value at which the slope of *M*_*a*_ changes is lowered as *T* increases. To investigate the influence of anisotropic *M* on transport, we measured the magnetoresistance, MR = $\frac{R\left(H\right)-R\left(0\right)}{R\left(0\right)}$, for the *c-* and *a*-axes (Figs. 1c and d), where $R\left(H\right)$ is the longitudinal resistance in *H*. While MR_*a*_ (MR with *H* along the *a* axis) was found to decrease monotonously upon increasing *H*, the value of MR_*c*_ (MR with *H* along the *c* axis) demonstrated an abrupt drop at *H*_flip_. The observed negative linear slope of MR_*c*_ above *H*_flip_ can be attributed to the magnon-MR (MMR) resulting from the electron-magnon scattering^40–42^. The negative slope of the MR is progressively increased as *T* rises (Fig. 1c in the main manuscript), consistent with the increase of magnon population^43^ and it is maximized at *T*_N_ (see Supplementary Fig. S4). The reproducibility of the MR measurement was tested using several different crystals (see Supplementary Fig. S5). Such close correspondence between the *M* and MR plots suggests that a magnetic order dominates the magneto-transport and its anisotropy.

**Figure 2:**
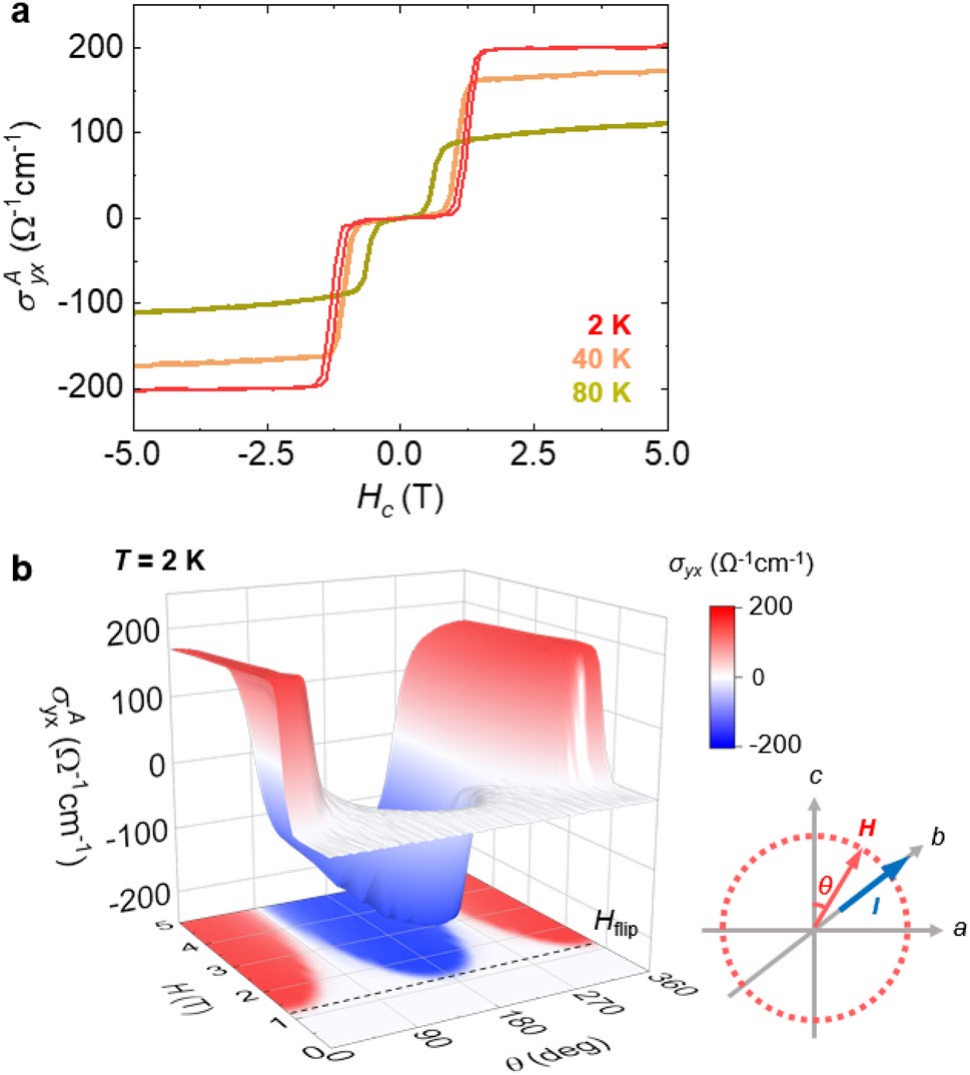
Large anomalous Hall effect. (**a**) *H* dependence of anomalous Hall conductivity, ${\sigma }_{yx}^{A}$, at *T* = 2, 40, and 80 K. (**b)** 2D and 3D contour plots of ${\sigma }_{yx}^{A}$, established from the angle dependence of ${\sigma }_{yx}^{A}$ for various *H* values at *T* = 2 K. For ${\sigma }_{yx}^{A}$, *H* is rotated in the *ac* plane with the current acting along the *b*-axis, *I//b*. The geometry of the Hall conductivity measurement is schematically illustrated: *θ* = 0° for the *c*-axis and *θ* = 90° for the *a*-axis. The grey dotted line denotes the spin-flip transition, *H*_flip_ = 1.2 T at 2 K.

The electrical Hall effect can be largely improved via the interplay between conduction electrons and magnetism. In ferromagnets, spin–orbit coupling provides an extra contribution to the ordinary Hall effect (known as anomalous Hall effect (AHE)), which has been identified to be proportional to *M*. In non-collinear antiferromagnets, a large AHE has been observed, despite the vanishingly small magnitude of *M*^44,45^. This AHE originates from the non-zero Berry curvature associated with topologically non-trivial spin textures^46,47^. In our collinear AFM CSCA crystal with a strong magnetocrystalline anisotropy, a certain value of *H* accompanies a spin-flip transition involving a drastic change in *M*. This spin-reorientation feature can be monitored via the AHE resulting from considerable spin–orbit coupling in the CSCA. Figure 2a illustrates that the transverse conductivity, defined as ${\sigma }_{yx}^{A}=\frac{{\rho }_{xy}}{{\rho }_{yy}^{2}+{\rho }_{yx}^{2}}$, directly follows the variation in *M* after the subtraction of a small linear component originating from the ordinary Hall effect (see Supplementary S2). We note that $x$, $y$, and $z$ correspond to $a$, $b$, and $c$, respectively. Here, ${\rho }_{yx}$ is the transverse resisitivity with the current acting along the *b* axis (*I//b*), ${\rho }_{yx}=-{\rho }_{xy}$, and ${\rho }_{yy}$ is the longitudinal resisitivity with *I//b*. The maximum ${\sigma }_{yx}^{A}$ ~200 Ω^-1^ cm^-1^ with a maximum anomalous Hall angle, *Θ*_AH_ = $\Delta {\sigma }_{yx}^{A}/{\sigma }_{yy}$ ≈ 2.24%, where ${\sigma }_{yy}$ is the longitudinal conductivity with *I//b*, was measured at 2 K, which reduced to its half magnitude at 80 K. ${\sigma }_{yx}^{A}$ can be well scaled by *M* with a scaling factor ${S}_{H}=$ ${\sigma }_{yx}^{A}/M$ ≈ 0.214 ${\mathrm{V}}^{-1}$ at 2 K and 0.156 ${\mathrm{V}}^{-1}$ at 80 K. The plot of *Θ*_AH_ and ${S}_{H}$ for various antiferromagnets and ferromagnets to compare with those in the CSCA is shown in Supplementary Fig. S7. Note that the *Θ*_AH_ and ${S}_{H}$ for the antiferromagnets were normalized to the magnetization in the flipped state. A direct comparison between ${\sigma }_{yx}^{A}$ and *M*_*c*_ at *T* = 2, 40, and 80 K is displayed in Supplementary Fig. S8. Figure 2b displays contour plots of ${\sigma }_{yx}^{A}$ obtained from the angular dependence of ${\sigma }_{yx}^{A}$ for various *H* values at 2 K, which clarifies the spin-flip driven emergence of the large AHE.

**Figure 3:**
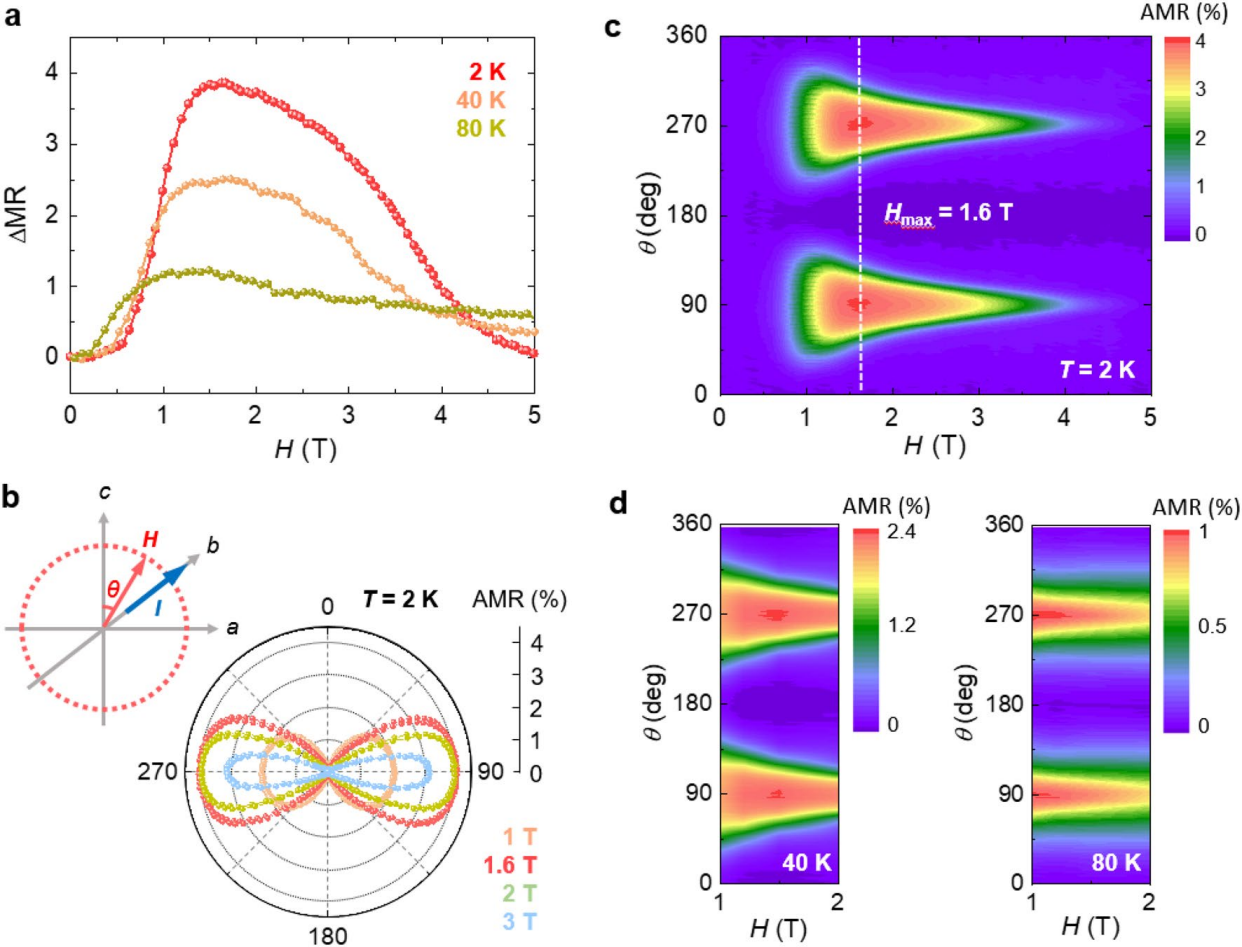
Anisotropic magnetoresistance. **(a)** MR difference between the *a-* and *c*-axes, ΔMR = MR_*a*_ – MR_*c*_, considered at *T* = 2, 40, and 80 K. (**b)** Polar angular plot of the anisotropic magnetoresistance (AMR), measured at *T* = 2 K by rotating *H* = 1, 1.6, 2, and 3 T in the *ac* plane with the current along the *b*-axis, *I//b*. The geometry of the AMR measurement is schematically shown: *θ* = 0° for the *c*-axis and *θ* = 90° for the *a*-axis. **(c)** Contour plot of the AMR measured at *T* = 2 K. (**d)** Contour plots of the AMR measured at *T* = 40 and 80 K.

A distinct MR difference can be demonstrated by plotting ΔMR = MR_*a*_ – MR_*c*_ as a function of *H*, as shown in Fig. 3a. A steep variation develops in the spin-flip regime, and the maximal difference becomes noticeable just after the occurrence of spin-flips at *H*_max_ ≈ 1.6 T for *T* = 2 K. Similar features accompanying the reduced ΔMR and lowered *H*_max_ can be observed at *T* = 40 and 80 K. The AMR, defined as $\frac{R\left(\theta \right)-R\left(0\right)}{R\left(0\right)}$, was measured with the geometry illustrated in Fig. 3b. Note that *H* is continually applied perpendicular to the current, inhibiting the ordinary Lorentzian MR effect. The uniaxial anisotropy allows for a two-fold rotational symmetry, which is presented in the polar angular plot of the AMR (Fig. 3b). The AMR reveals a dumbbell-like shape in which the maximum value occurs at *θ* = 90 and 270°. Across *H*_flip_, the AMR is considerably enhanced, and the largest variation occurs at *H*_max_ = 1.6 T, which is consistent with ΔMR. The maximum AMR at *H*_max_ reaches ~ 4%, which is one order of magnitude larger than the corresponding value for other AFM metals^6,48,49^. The complete AMR contour map in Fig. 3c reveals that the AMR effect is evidently maximized at *H*_max_, above which the AMR is gradually reduced owing to the weakened anisotropy. The decrease in *H*_max_ is displayed in the contour plots at *T* = 40 and 80 K (Fig. 3d), corroborating with the ΔMR behavior (Fig. 3a). Detailed *T* evolution of the AMR effect is presented in the *θ–T* contour plots in Supplementary Fig. S9.

**Figure 4:**
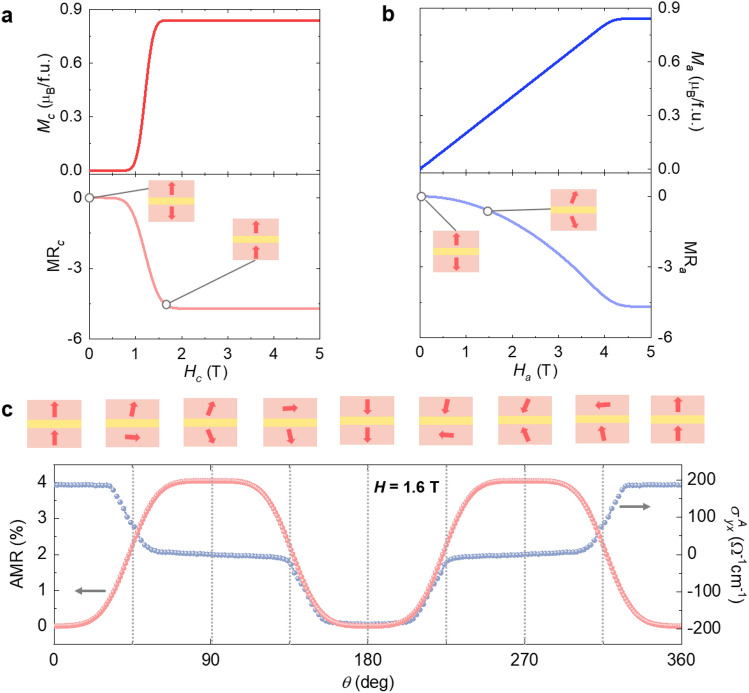
Intrinsic magnetic-multilayer structure and electrically-detectable spin states. (**a**) Calculated isothermal *M*_*c*_ and MR_*c*_. The schematic layered structure presents two ferromagnetic (FM) Co layers with relatively oriented net moments for a specific value of *H*, separated by a nonmagnetic (NM) Ca/Sr layer in a unit cell. (**b)** Calculated isothermal *M*_*a*_ and MR_*a*_. **c** The AMR was obtained by conductance calculation for *H*_max_ = 1.6 T. The experimental result of angular dependent ${\sigma }_{yx}^{A}$ taken at 2 K for *H*_max_. Schematics present relative orientations of the net magnetic moments in a unit cell with respect to the angle variation in *H*, which is estimated by an easy-axis magnetocrystalline anisotropy model.

To clarify the nature of the magnetic phase transition, theoretical calculations based on the spin Hamiltonian with uniaxial magnetocrystalline anisotropy were performed. Upon increasing *H* along the magnetically easy *c-*axis, the magnetic energies of the AFM and spin-flip phases are presented as ${E}_{\mathrm{AFM}}=-2J{S}^{2}$ and ${E}_{\mathrm{flip}}=2J{S}^{2}-2g{\mu }_{\mathrm{B}}HS,$ respectively. Here, $J$ represents the AFM coupling strength, $g$ = 2, and $S$ represents the net moment of the Co_2_As_2_ layers. Employing the condition ${E}_{\mathrm{AFM}}={E}_{\mathrm{flip}}$ at *H*_flip_, the following relation $g{\mu }_{B}{H}_{\mathrm{flip}}/JS=2$ is obtained. Further, we theoretically calculate the magnetization *M*_*c*_ as a function of *H* by minimizing the spin Hamiltonian in the presence of *H* along the magnetic easy *c-*axis. An estimation of $J{S}^{2}=5.66\times {10}^{4} \mathrm{J}/{\mathrm{m}}^{3}$ is obtained by fitting the theoretical result for *M*_*c*_ to the experimental data recorded at 2 K, consistent with our previous work^25^. Following a similar analysis in the presence of *H* along the *a-*axis, we also construct the magnetization *M*_*a*_ as a function of *H*. By fitting the theoretical result for *M*_*a*_ to the experimental data recorded at 2 K, we estimate the magnetocrystalline anisotropy constant $K=1.4 J{S}^{2}$^25^. The foregoing belongs to strong magnetocrystalline anisotropy, as $K>J{S}^{2}$ for our model Hamiltonian. However, at the *H*-induced transition in CSCA, the experimentally observed step-like increase does not reach its saturated state, primarily owing to two reasons. First, a non-linear regime appears just after the large step-like increase (Fig. 1c) owing to spatial modulations of the phase coexistence pertaining to the first-order nature of the transition^50^. By considering the spatial modulations, we present the resulting *M*_*c*_ and* M*_*a*_ in Figs. 4a and b, which can be observed to conform with the experimental data (Fig. 1c and d). Second, a slight linear slope can be observed above the nonlinear regime, which increases continually as *T* increases, with the reduction in the *M*_*c*_ value at the largest measured *H* (Fig. 1c). These observations suggest that the slope is attributed to the thermal fluctuation inhibiting the saturation of *M*_*c*_.

The essential influence of the strong magnetocrystalline anisotropy, found in the magnetic response of net magnetic moments, can explain the occurrence of a spin-flip transition. The schematic layered structure in Fig. 4a represents two FM Co layers with relatively oriented net moments, which are split by an NM Ca/Sr layer in a unit cell. The magnetocrystalline anisotropy tends to bind spin directions to a specific crystal axis. Consequently, the AFM phase of CSCA in the presence of zero *H* is stabilized along the *c*-axis, with a minimized total magnetic energy. *H* acting along the *c-*axis (*H*_*c*_) activates the spin-flips, accompanied by a large increase in *M*_*c*_, i.e., flips in the net moments. This situation is akin to a giant magnetoresistance-type (GMR-type) device that is artificially constructed by magnetic layers. The GMR effect has been observed in two geometries: current applied in the plane of the layers (CIP) and current applied perpendicular to the plane of the layers (CPP)^51^. The MR with CPP is found to be definitely larger than that with CIP, which is ascribed to the different scaling lengths. This description persists in the CIP and CPP geometries as long as the thickness of the layer is smaller than the scaling length. We note that due to the geometry of very thin CSCA crystals, no measurable MR or AMR with the current acting along the *c* axis (*I//c*) was detected within the accuracy of our resistivity measurement. In contrast, *H*_*a*_ generates steadily canted net moments, which is a fingerprint of a highly anisotropic nature (Fig. 4b).

Since the MR shows a noticeable dependence on the magnetic structure varied by external magnetic field, one can presume that electron hopping between adjacent Co_2_As_2_ layers is dominated by spin-independent part. For the spin-dependent part, the electron transport can be well described by hopping through double exchange mechanism mediated by a non-magnetic Ca/Sr intermediate layer. To theoretically determine the magneto-transport property, we assume that the interlayer hopping amplitude is given by ${t}_{i, i+1}= \left|\langle {\widehat{n}}_{i}|{\widehat{n}}_{i+1}\rangle \right|$ (*i* = 1, 2). Here $\left|\langle {\widehat{n}}_{i}|{\widehat{n}}_{i+1}\rangle \right|$ denotes the overlap integral between two spinors, where each spinor is aligned parallel to the spins in each layer. It is described by $\mathrm{cos}\frac{\gamma }{2}$, where $\gamma$ represents the relative angle between two spinors. For the GMR-type geometries, the spin-dependent conductivity was described by utilizing a transfer integral between the nearest-neighbor layers^52^. The layer dependent conductivity is given by Green functions between the adjacent magnetic layers, which incorporate the transfer matrix element, $t$. Considering the Green functions, the conductance of our system can be assumed to be proportional to multiple hopping amplitudes through the bilayer geometry, and hence, $\sigma \propto {t}_{12}{t}_{23}\propto {\mathrm{cos}}^{2}\gamma /2$. From the definition of MR and $R=1/\sigma$, we can infer that MR is proportional to $\sigma \left(0\right)-\sigma \left(H\right)$. The development of anisotropic magneto-transport properties through a flip transition can be reasonably described by $\sigma$ calculated directly from relative spin orientations between different Co_2_As_2_ layers, as depicted in Fig. 4a and b.

Conduction electrons are crucial in tracking the various spin states formed in the presence of rotating *H*_max_ = 1.6 T, in the magnetic-multilayer structure. Figure 4c depicts the AMR and angular dependence of AHE at *H*_max_. Combined with theoretical calculations, the different spin states are electrically probed, as shown in the schematics. The various spin states are clearly discernible for the AMR and angular dependence of AHE at *H*_max_. With the rotation of *H* from 0° to 90°, the net parallel moments convert to an antiparallel arrangement by switching the net moment in one of the two layers, which transforms a low *R* state to a high *R* state or a positive ${\sigma }_{yx}^{A}$ state to a zero ${\sigma }_{yx}^{A}$ state. Further rotation from 90° to 180° induces the high to low *R* or zero to negative ${\sigma }_{yx}^{A}$ switching by orienting the net moment in the other of the two layers. The AHE apparently satisfies the Onsager relation: ${\sigma }_{yx}^{A}\left(\overrightarrow{H}\right)=-{\sigma }_{yx}^{A}\left(-\overrightarrow{H}\right).$

## Conclusion

In summary, herein, we investigate anisotropic magnetic properties in a Ising-type layered antiferromagnet, Ca_0.9_Sr_0.1_Co_2_As_2_. This intrinsic magnetic-multilayer structure reveals the spin-flip-driven large anomalous Hall conductivity. In addition, the switching operation between low and high resistance states is demonstrated in the presence of a rotating magnetic field. This leads to anisotropic magnetoresistance, which is maximized in the vicinity of the flip transition. Our theoretical estimation verifies that the easy-axis magnetocrystalline anisotropy is a key factor for the observed anisotropic magnetic properties. Electrical detection for diverse spin states is applicable to a wide range of antiferromagnets.

## Methods

The Ca_0.9_Sr_0.1_Co_2_As_2_ single crystals were grown via the self-flux method^22,53^. A CoAs precursor was first prepared via a solid-state reaction using mixed powders of Co (99.5%, Alfa Aesar) and As (99,999%, Sigma Aldrich) with a fixed stoichiometric ratio, after which the mixture was calcined in air at 700 °C for 24 h in a furnace. The CoAs powder was blended with Ca and Sr flakes at a CoAs:Ca:Sr molar ratio of 4:0.9:0.1, and the mixture was transferred into an alumina crucible. The crucible was vacuum-sealed in a quartz tube. In a high-temperature furnace, the quartz tube was heated at 1280 °C for 16 h, slowly cooled to 850 °C at a rate of 2 °C/h, and then cooled to room temperature at a rate of 100 °C/h. Finally, crystals with typical dimensions of 1.5 × 3 × 0.2 mm^3^ were obtained.

Samples for STEM analysis were prepared with a cutting plane perpendicular to the *a*-axis using a dual-beam focused ion beam system (Helios 650, FEI). The cutting plane displayed a well-identifiable atomic structure. To avoid critical damage to thin samples, acceleration voltage conditions were lowered from 30 to 2 keV. The atomic structure was investigated via STEM (JEM-ARM200F, JEOL Ltd, Japan) at 200 keV with a Cs-corrector (CESCOR, CEOS GmbH, Germany) and cold field emission gun. The size of the electron probe was 83 pm, and the range of the high-angle annular dark-field detector angle was 90–370 mrad.

Magnetic susceptibility and isothermal magnetization measurements were carried out with magnetic fields acting along *a*- and *c*-axes using a vibrating sample magnetometer module of a physical property measurement system (PPMS, Quantum Design, Inc.). The analyses for determining the magnetic-field dependences of the in-plane resistivity and Hall resistivity were carried out using a conventional four-probe configuration in the PPMS. A description pertaining to the electrical contacts is included in Supplementary Note 2. The anisotropic magnetoresistance and angle dependence of the anomalous Hall resistivity were obtained via a polar angle scan of the magnetic fields in the *ac* plane of the PPMS equipped with a single-axis rotator.

The spin Hamiltonian with uniaxial magnetocrystalline anisotropy can be expressed as

$$ H/N = J\sum\nolimits_{(i = 1)}^{2} {\vec{S}_{i} \cdot } \vec{S}_{(i + 1)} - g\mu_{B} \vec{H} \cdot \sum\nolimits_{(i = 1)}^{2} {\vec{S}_{i} } + K\sum\nolimits_{(i = 1)}^{2} {{\text{sin}}^{2} \theta_{i} } , $$

where $J$ represents the AFM coupling strength between Co moments in adjacent layers, $N$ denotes the number of Co moments in a single layer, $g$ = 2, and $K$ denotes the magnetocrystalline anisotropy constant. The second term represents the Zeeman energy, where the magnetic field $\overrightarrow{H}$ acts in the *ac* plane making an angle *θ* with the *c*-axis. The third term denotes the uniaxial magnetocrystalline anisotropy energy, which is consistent with the favorable spin orientation along the *c*-axis.

## Supplementary Information

Supplementary Information.

## Data Availability

The data that support the findings of this study are available from the corresponding authors upon reasonable request.
