# Supplementary material for: Spin-flip-driven anomalous Hall effect and anisotropic magnetoresistance in a layered Ising antiferromagnet
Source: Sci Rep. 2023 Feb 28;13:3391. doi: 10.1038/s41598-023-30076-2 (PMC9974960; doi:10.1038/s41598-023-30076-2)
Supplement: Supplementary file 1 — Supplementary Information. [file 41598_2023_30076_MOESM1_ESM.pdf]

Supplementary Information

Spin-flip-driven anomalous Hall effect and anisotropic magnetoresistance in a layered Ising antiferromagnet

Dong Gun Oh[1,†], Jong Hyuk Kim[1,†], Mi Kyung Kim[1], Ki Won Jeong[1], Hyun Jun Shin[1], Jae Min Hong[1], Jin Seok Kim[1], Kyungsun Moon[1], Nara Lee[1,*], and Young Jai Choi[1,*]

[1]Department of Physics, Yonsei University, Seoul 03722, Korea.

[†]These authors contributed equally: D. G. Oh, J. H. Kim.

*email: eland@yonsei.ac.kr; phylove@yonsei.ac.kr

Supplementary S1. Basic characterizations of $Ca_{0.9}Sr_{0.1}Co_2As_2$ (CSCA) crystals

The crystallographic structure of CSCA was inspected via single-crystal X-ray diffraction (SC-XRD) analysis performed on a Rigaku XtaLAB PRO with a Mo K_α source (λ = 0.71073 Å) at 93 K. Accordingly, a small and clean piece of the plate-shaped crystal was chosen for the SC-XRD analysis. The CrysAlisPro software was used for data acquisition and processing. In Figs. S1a–S1c, collapsed SC-XRD data are presented for the reciprocal (0kl), (h0l), and (hk0) scattering planes, respectively. The four-fold rotational symmetry surrounding the reciprocal *l*-axis is depicted in Supplementary Fig. S1c. With the application of SHELXL[1], the structure of CSCA was refined to a tetragonal structure with space group *I*4/*mmm* (No. 139). The goodness parameters were consequently obtained from the refinement as R_1 = 0.0286, wR_2 = 0.0779, and *GooF* = 1.132. The lattice parameters are defined as $a = b$ = 0.39970(2) nm and c = 1.03356(10) nm at 93 K, and the detailed refinement results are listed in Table S1–S3.

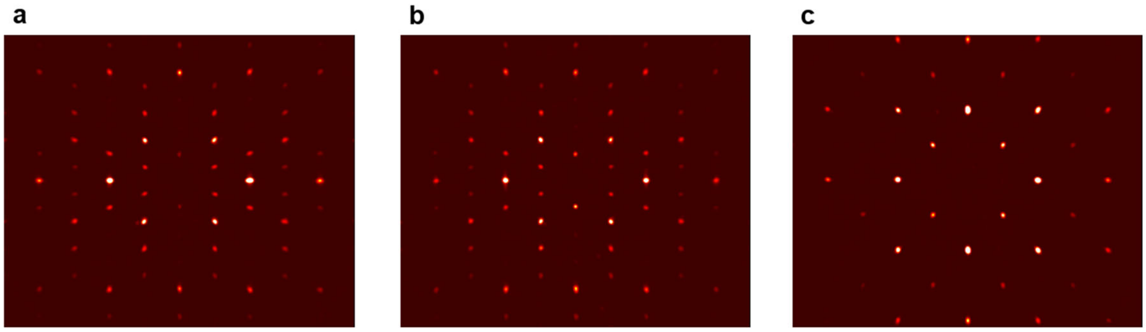

Fig. S1 Laue scattering patterns. Single-crystal X-ray diffraction data for **a** (0kl), **b** (h0l), and **c** (hk0) scattering planes. CSCA crystallizes in a body-centered tetragonal structure with space group *I*4/*mmm* (No. 139).

Table S1. Crystallographic and structural refinement results of CSCA obtained from single-crystal X-ray diffraction data recorded at 93 K.

Crystal system	Tetragonal
Space group	*I*4/*mmm* (No. 139)
Lattice parameter	$a = b = 0.39970(2)$ nm
	$c = 1.03356(10)$ nm
	$\alpha = \beta = \gamma = 90°$
Volume	165.13(2) $Å^3$
Formula units	$Z = 2$
Density	6.286 g/cm^3
Temperature	93 K
Radiation	Mo K_α
Wavelength	$\lambda = 0.71073$ Å
Linear absorption coefficient	$\mu = 32.631$ mm^{-1}
F(000)	284
Total/unique reflections	4071/94
Number of parameters/restraints	8/0
Range for data collection	$-5 \leq h \leq 5$, $-5 \leq k \leq 5$, $-14 \leq l \leq 13$
	$3.943° \leq \theta \leq 29.952°$
Reflections threshold	$I > 2\sigma (I)$

R indexes of merging	R_{int} = 0.0789, R_σ = 0.0148
Final R indexes for all 102 data	R_1 = 0.0286, wR_2 = 0.0779
Goodness of fit	*GooF* = 1.132
Largest difference peak/hole	1.701 e Å^{-3}/-1.517 e Å^{-3}

Table S2. Refined atomic positions of CSCA obtained from single-crystal X-ray diffraction data.

Atom	Wyckoff	x	y	z	occupancy
Ca	2b	1	1	0.5	0.877(18)
Sr	2b	1	1	0.5	0.123(18)
Co	4d	0.5	0	0.25	1
As	4e	0.5	0.5	0.36723(9)	1

Table S3. Anisotropic and equivalent/isotropic thermal displacement parameters for the atoms of CSCA. U_{eq} is defined as one third of the trace of the orthogonalized U_{ij} tensor. For all atoms, $U_{12} = U_{23} = U_{31} = 0$. All parameters are given in Å^2.

Atom	U_{11}	U_{22}	U_{33}	U_{eq}
Ca	0.0049(10)	0.0049(10)	0.0075(13)	0.0057(9)
Sr	0.0049(10)	0.0049(10)	0.0075(13)	0.0057(9)
Co	0.0056(5)	0.0056(5)	0.0090(7)	0.0067(4)
As	0.0042(4)	0.0042(4)	0.0087(6)	0.0057(4)

Magnetic susceptibility, $\chi = M/H$, measured at H = 0.1 T upon warming after zero-field-cooling, indicates the emergence of antiferromagnetic (AFM) order at $T_N \approx 97$ K, as plotted in Fig. S2a. Magnetic measurements were carried out with magnetic fields along *a*- and *c*-axes using a vibrating sample magnetometer module of a physical property measurement system (PPMS, Quantum Design, Inc.). The anisotropic nature manifested in the χ curves for H along the *a*- and *c*-axes. The rapid decrease in χ below T_N along the *c*-axis is consistent with the alignment

of magnetic moments of Co ions along this axis. Electric transport measurements were carried out at zero H in the PPMS. The resistivity as a function of T was found to exhibit metallic behavior and a distinct anomaly at T_N identified in its T derivative (Fig. S2b).

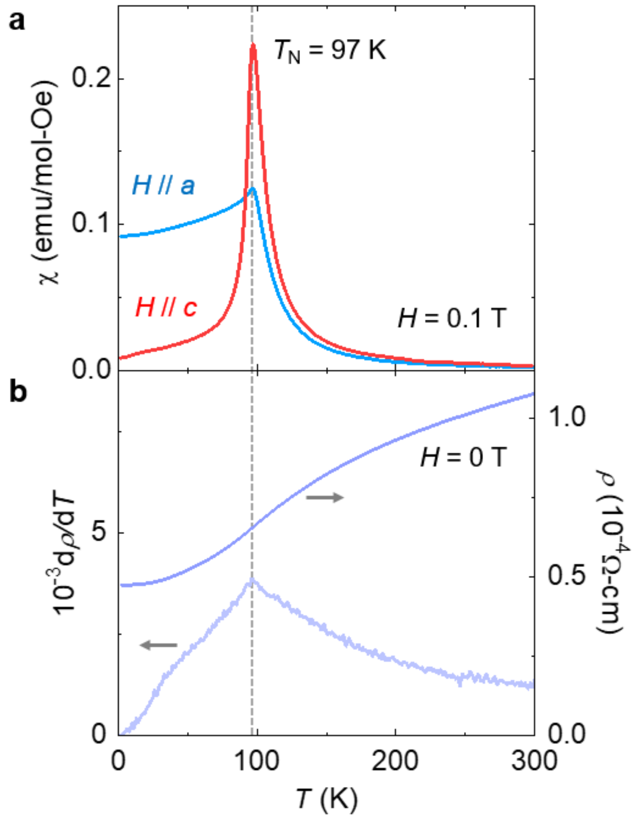

Fig. S2 Temperature dependence of antiferromagnetic properties. a Temperature dependence of the magnetic susceptibility, $\chi = M/H$, measured upon warming at $H = 0.1$ T after zero-field cooling along the a- and c-axes. The vertical grey line indicates the Néel temperature, $T_N = 97$ K. **b** Temperature dependence of resistivity at a zero magnetic field and its temperature derivative.

Supplementary S2. Analyses of physical properties

As the CSCA is a layered magnetic material, it possesses the (001) cleavage. To determine the longitudinal (MR), transverse (AHE), and angle-dependent (AMR) electrical transports, we prepared bulk single-crystalline samples with the cleaved (001) plane. As displayed in Fig. S3, gold wires of 25 μm are attached to a 20 μm-thick cleaved (001) plane of the CSCA crystal

using a silver conductive epoxy (H20E, EPO-TEK®).

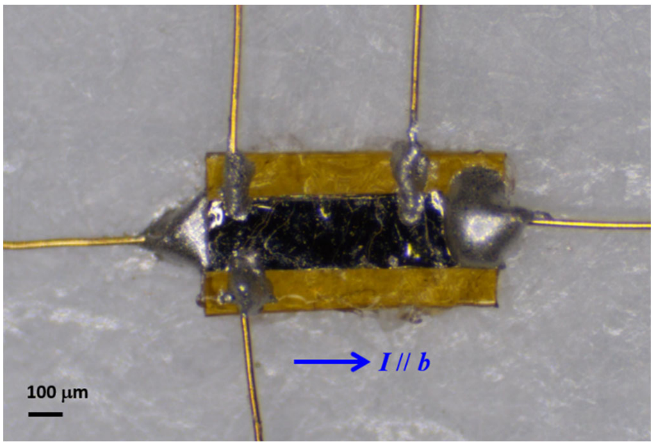

Fig. S3 Image of a CSCA crystal with electrical contacts. Optical microscopic image of four-point probes attached on a cleaved CSCA crystal for MR, AMR, and Hall resistivity measurements. The electric current is applied along the *b*-axis.

In Fig. S4, the temperature dependence of the negative slope of the MRc is plotted. The result shows a progressive increase as T rises. The slope appears to be maximized at T_N, followed by the gradual decrease above T_N.

Figure S5a displays the error bars obtained from the spin-flip transitions at various temperatures. For the measurement of temperature dependent MR and AHE, the same sample was used for consistency. Over the time scale of these measurements, no deterioration was observed. To verify the reproducibility of our measurements, several different samples from the same batch were also tested by analyzing their physical properties. For example, the MR data recorded at 2 K for different crystals (from sample 1 (S1) to sample 4 (S4)) are all consistent, as illustrated in Fig. S5b.

The original data obtained from the Hall measurement are presented in Fig. S6a. The linear Hall coefficient originating from the ordinary Hall effect is estimated as $R_H = -2.4 \times 10^{-10}$ m^3/C.

The anomalous Hall conductivity obtained from background subtraction is plotted in Supplementary Fig. S6b.

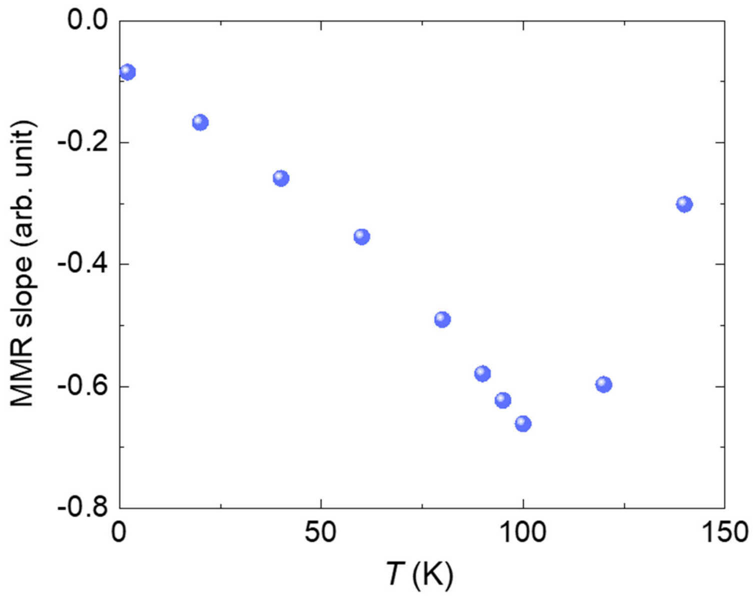

Fig. S4 Slope of MMR. Temperature dependence of MMR slope taken from the MR_c at various temperatures.

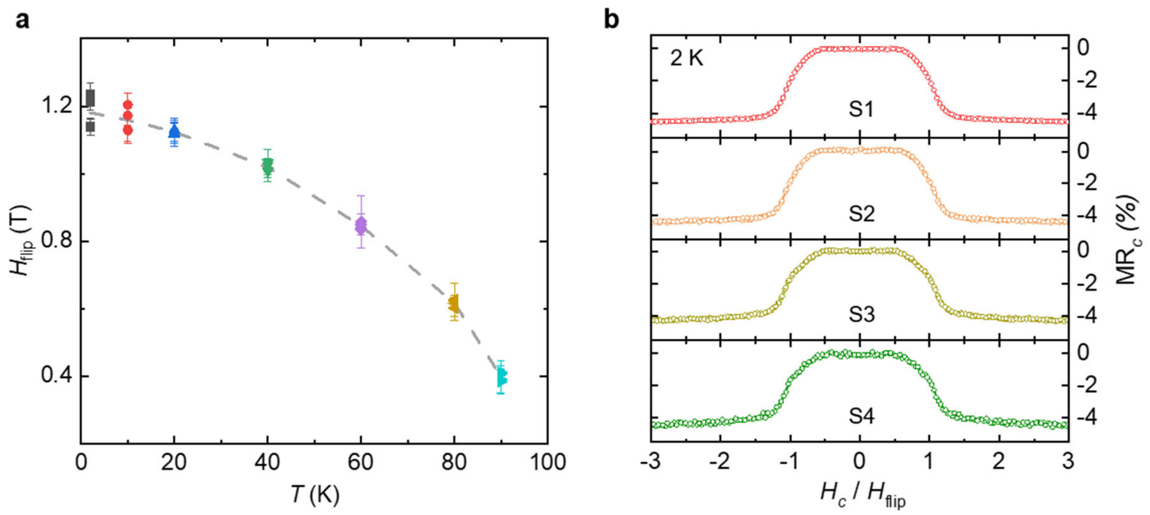

Fig. S5 Error bars of spin-flip transitions and magnetoresistance of different samples. a The error bars of spin-flip transitions at T = 2, 10, 20, 40, 60, 80, and 90 K. **b** Magnetoresistance along the c-axis, MR_c, at 2 K for four different CSCA crystals (S1-S4).

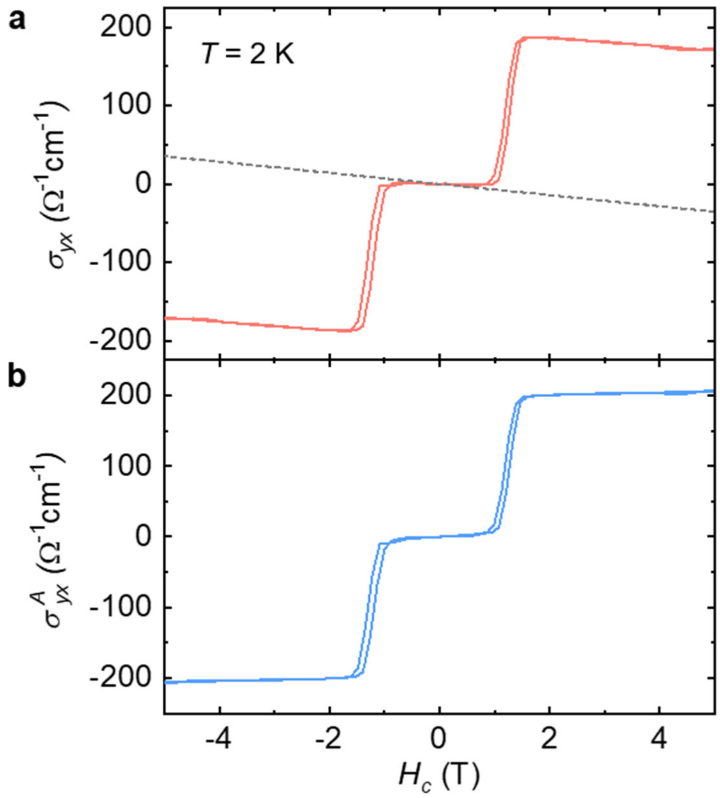

Fig. S6 Anomalous Hall conductivity at 2 K a Hall conductivity measured at 2 K. The grey dashed line indicates the component of the ordinary Hall effect. **b** Anomalous Hall conductivity at 2 K obtained after subtracting the ordinary Hall component.

The intimate correlation between the transverse conductivity and magnetization is evidenced in the scaling factor, $S_H = \sigma_{yx}^A/M$. The anomalous Hall angle, defined as $\Theta_{\mathrm{AH}} = \Delta\sigma_{yx}/\sigma_{xx}$, indicates the deviation of the electron flow from the current direction. These parameters, are known to be key elements to evaluate the capacity and compatibility of a Hall device. The values estimated from the spin-flip-driven AHE in CSCA turn out to be relatively large in comparison with those in antiferromagnetic (Mn_3Sn, $MnBi_2Te_4$, $MnBi_4Te_7$, and $Mn(Bi_{1-x}Sb_x)_2Te_4$ (x = 0.79)) and ferromagnetic (Fe_4GeTe_2, $Mn_5Si_3C_{0.8}$, MnSi, Mn_5Ge_3, $Fe_{1/4}TaS_2$, Fe_3Sn_2, and Cr-$CrTe_2$) metals, as shown in Fig. S7[2-10].

In Fig S8, to facilitate comparison, the anomalous Hall conductivity and magnetization with magnetic field along the c-axis at T = 2, 40, and 80 K are presented in the same figure. These

electrical and magnetic properties appear well compatible, suggesting that conduction electrons can be utilized to probe magnetic properties.

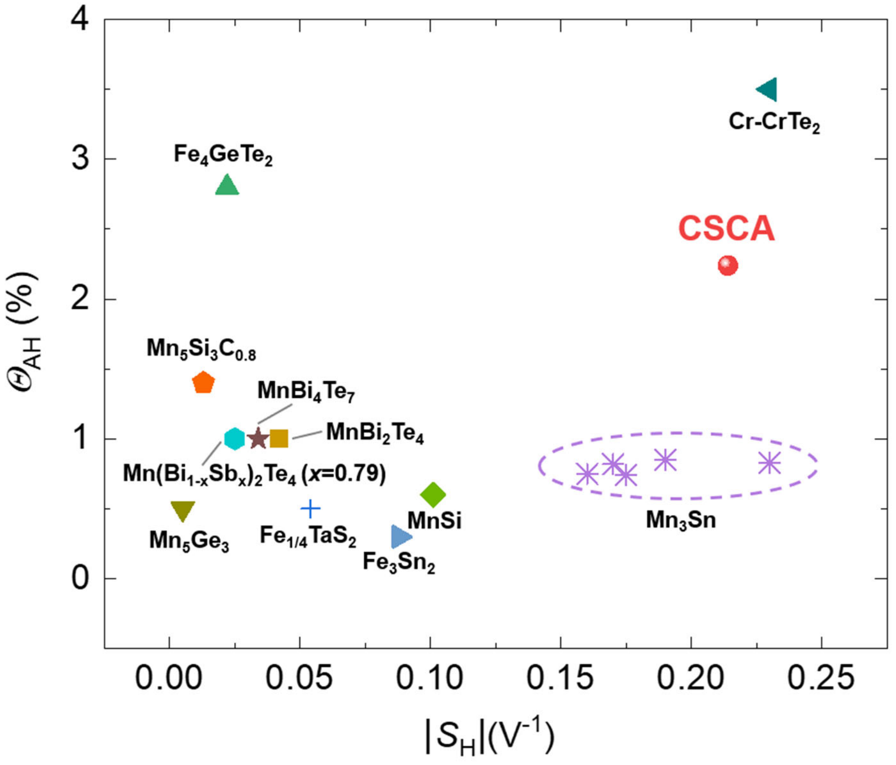

Fig. S7 Anomalous Hall angle and anomalous Hall factor of CSCA and metallic ferromagnets. Plot of anomalous Hall angle, defined as $\Theta_{\mathrm{AH}} = \Delta\sigma_{yx}/\sigma_{xx}$, and scaling factor, $S_H = \sigma_{yx}^{A}/M$ for various metallic antiferromagnets and ferromagnets, in comparison with those of the CSCA, which are associated with the net magnetization formed above the spin-flip transition.

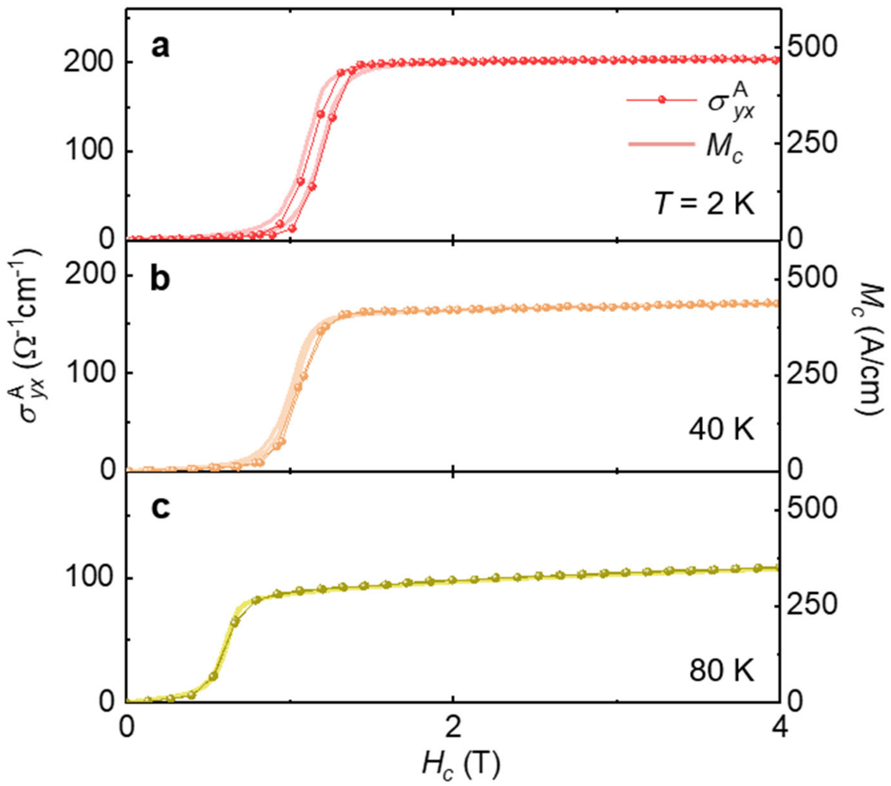

Fig. S8 Comparison between the anomalous Hall conductivity and magnetization. a H_c dependence of the anomalous Hall conductivity (closed circles) and magnetization (solid curves) at T = 2 K. **b** H_c dependence of the anomalous Hall conductivity (closed circles) and magnetization (solid curves) at T = 40 K. **c** H_c dependence of the anomalous Hall conductivity (closed circles) and magnetization (solid curves) at T = 80 K.

S3. Temperature evolution of anisotropic magnetoresistance

During our analysis, we examined the T evolution of AMR = $\frac{R(\theta)-R(0)}{R(0)}$, measured in the geometry presented in Fig. S8a. The AMR comprises two different components: A noncrystalline component possibly arising from the relative orientation between M and a specific current direction, and a crystalline component entirely attributed to the crystal symmetry[11-13]. The conventional AMR effect in polycrystalline 3d ferromagnetic alloys is ascribed predominantly to the noncrystalline AMR component, as all crystalline components are averaged out. In an attempt to realize the AMR effect driven dominantly by magnetocrystalline anisotropy in AFM spintronics, the crystalline AMR component in single-

crystalline bulk and thin film materials has been particularly demonstrated[14,15]. The two-fold rotational symmetry is reflected in the polar angular plot of the AMR measured at H_{max} = 1.6 T (Fig. S9a). The dumbbell-like shape, indicating uniaxial magnetocrystalline anisotropy, diminishes progressively as *T* increases to 80 K. Detailed *T* dependence of the AMR is plotted in the *θ*–*T* contour plot (Fig. S9b). A smaller magnitude but broader shape of the AMR can be observed at *H* = 1 T (Fig. S9c). At *H* = 3 T, the narrower shape of the AMR almost disappears around 60 K (Fig. S9d).

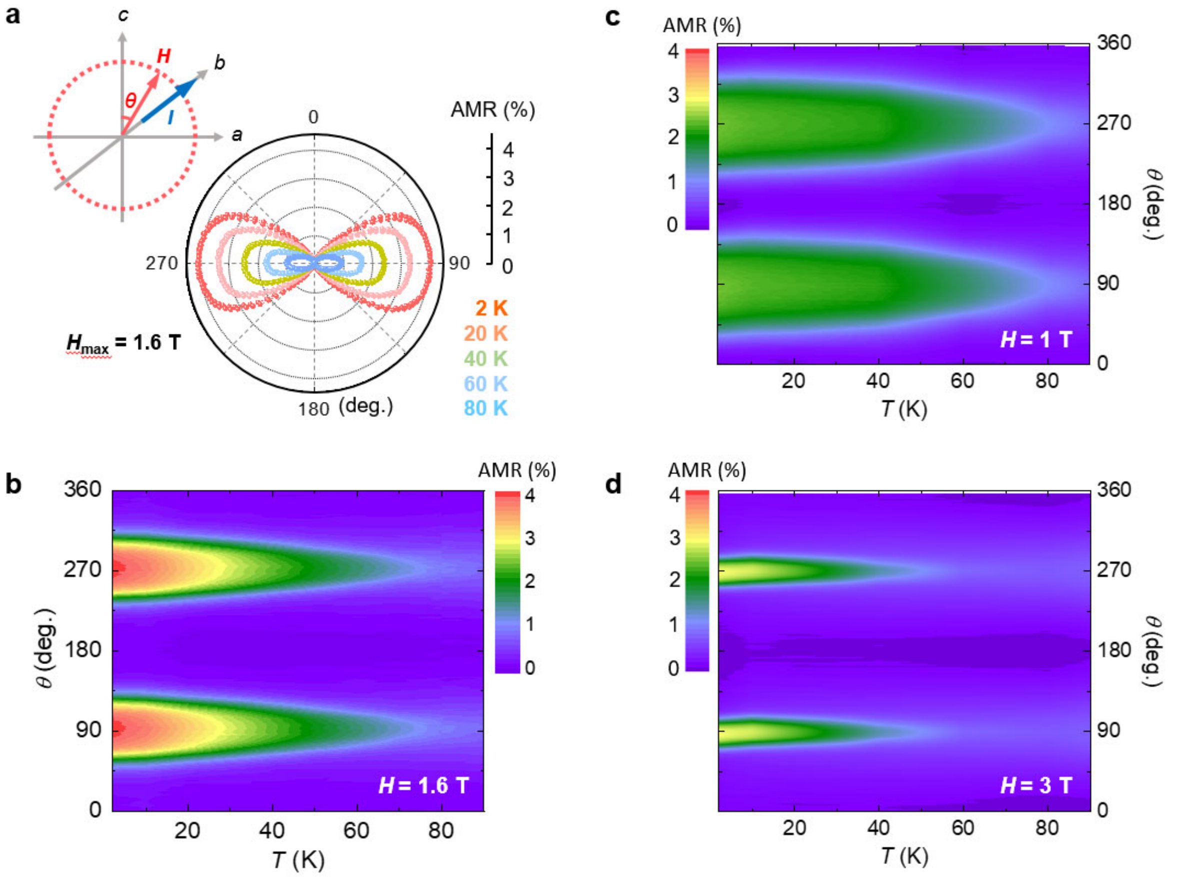

Fig. S9 Temperature evolution of anisotropic magnetoresistance. **a** Geometry for the AMR measurement is schematically shown. *H* is rotated in the *ac* plane, while the current is applied along the *b*-axis, *I*//*b*. Polar angular plot of the AMR, measured at *T* = 2, 20, 40, 60, and 80 K by rotating H_{max} =1.6 T. **b** *θ*–*T* contour plot constructed from the AMR data recorded at various values of *T* and H_{max} =1.6 T. **c-d** *θ*–*T* contour plots of the AMR for *H* = 1 and 3 T, respectively.

References

1. Hübschle, C. B., Sheldrick, G. M. & Dittrich, B. ShelXle: a Qt graphical user interface for SHELXL. *J. Appl. Crystallogr.* **44**, 1281-1284 (2011).

2. Checkelsky, J. G., Lee, M., Morosan, E., Cava, R. J. & Ong, N. P. Anomalous Hall effect and magnetoresistance in the layered ferromagnet $Fe_{1/4}TaS_2$: The inelastic regime. *Phys. Rev. B* **77**, 014433 (2008).

3. Lee, M., Onose, Y., Tokura, Y. & Ong, N. P. Hidden constant in the anomalous Hall effect of high-purity magnet MnSi. *Phys. Rev. B* **75**, 172403 (2007).

4. Huang, M. *et al.* Significant perpendicular magnetic anisotropy in room-temperature layered ferromagnet of Cr-intercalated $CrTe_2$. *2D Materials* **8**, 031003 (2021).

5. Sürgers, C., Fischer, G., Winkel, P. & Löhneysen, H. v. Magnetotransport in ferromagnetic Mn_5Ge_3, $Mn_5Ge_3C_{0.8}$, and $Mn_5Si_3C_{0.8}$ thin films. *Phys. Rev. B* **90**, 104421 (2014).

6. Wang, Q., Sun, S., Zhang, X., Pang, F. & Lei, H. Anomalous Hall effect in a ferromagnetic Fe_3Sn_2 single crystal with a geometrically frustrated Fe bilayer kagome lattice. *Phys. Rev. B* **94**, 075135 (2016).

7. Yan, J. *et al.* Room-temperature angular-dependent topological Hall effect in chiral antiferromagnetic Weyl semimetal Mn_3Sn. *Appl. Phys. Lett.* **115**, 102404 (2019).

8. Kim, K. *et al.* Large anomalous Hall current induced by topological nodal lines in a ferromagnetic van der Waals semimetal. *Nat. Mater.* **17**, 794-799 (2018).

9. Lee, S. H. *et al.* Evidence for a Magnetic-Field-Induced Ideal Type-II Weyl State in Antiferromagnetic Topological Insulator $Mn(Bi_{1-x}Sb_x)_2Te_4$. *Phys. Rev. X* **11**, 031032 (2021).

10. Hu, C. *et al.* A van der Waals antiferromagnetic topological insulator with weak interlayer magnetic coupling. *Nat. Commun.* **11**, 97 (2020).

11. Rushforth, A. W. *et al.* Anisotropic Magnetoresistance Components in (Ga,Mn)As. *Phys. Rev. Lett.* **99**, 147207 (2007).

12. Zeng, F. L. *et al.* Intrinsic Mechanism for Anisotropic Magnetoresistance and Experimental Confirmation in Co_xFe_{1-x} Single-Crystal Films. *Phys. Rev. Lett.* **125**, 097201 (2020).

13. McGuire, T. & Potter, R. Anisotropic magnetoresistance in ferromagnetic 3d alloys. *IEEE T. Magn.* **11**, 1018-1038 (1975).

14. Lee, N. *et al.* Antiferromagnet-Based Spintronic Functionality by Controlling Isospin Domains in a Layered Perovskite Iridate. *Adv. Mater.* **30**, 1805564 (2018).

15. Fina, I. *et al.* Anisotropic magnetoresistance in an antiferromagnetic semiconductor. *Nat. Commun.* **5**, 4671 (2014).
